# Cardiac resynchronization therapy in very old patients with pacemakers: a cardiogeriatric, physiology-based approach

**DOI:** 10.1007/s40520-026-03393-5

**Published:** 2026-04-11

**Authors:** Rémi Esser, Marc Harboun, Alejandro Mondragon, Marine Larbaneix, Christine Farges, Marlène Esteban, Vincenzo Palermo, Sophie Nisse Durgeat, Olivier Maurou, David Perrot

**Affiliations:** 1 Cardiogeriatric Department, Hôpital La Porte Verte, Versailles, France; 2 Cardiology Department, Hôpital Marie Lannelongue, Le Plessis Robinson, France; 3 Medical Affairs, NP Medical, Bordeaux, France; 4 Cardiology Department, Groupe Hospitalier Privé Ambroise Paré- Hartmann, Neuilly sur Seine, France

**Keywords:** Cardiac resynchronization therapy, Very old adults, Frailty, Pacemaker-induced cardiomyopathy, Geriatric cardiology, Quality of life

## Abstract

Cardiac resynchronization therapy (CRT) is an established treatment for selected patients with heart failure (HF), yet its role in advanced age remains insufficiently defined. Adults aged ≥ 80 years are markedly underrepresented in randomised trials, frequently present with multimorbidity and frailty, and often develop HF related to pacing-related mechanisms, including pacing-induced cardiomyopathy (with LVEF decline) and pacing-related dyssynchrony that is not fully addressed by guideline-based algorithms. In this context, CRT implantation or upgrade represents a complex cardiogeriatric decision rather than a purely electrophysiological intervention. This narrative review examines the physiological rationale, available evidence, and clinical challenges of CRT in advanced-age populations, illustrated by a representative clinical vignette of pacemaker-induced HF. We discuss age-related myocardial vulnerability, secondary mitral regurgitation, competing mortality risks, and the predominance of functional and quality-of-life outcomes over survival endpoints. Particular emphasis is placed on integrating geriatric domains—frailty, functional status, cognition, and proportionality of care—into CRT decision-making. We explicitly distinguish pacing-induced cardiomyopathy (with reduced LVEF) from isolated pacing-related dyssynchrony; in patients with preserved LVEF, congestion is more often driven by a severe haemodynamic substrate—particularly valve dysfunction—than by dyssynchrony alone. In selected older adults, CRT may provide meaningful symptomatic and functional benefit when HF is driven by potentially reversible dyssynchrony, even if survival gains are limited. A cardiogeriatric, physiology-based approach may help identify patients most likely to benefit from CRT while avoiding disproportionate interventions in individuals with advanced frailty or limited physiological reserve.

## Introduction

### Heart failure and ageing: a geriatric-dominated condition

Heart failure (HF) predominantly affects older adults and represents one of the leading causes of hospitalisation, disability and loss of autonomy in ageing populations. In this review, “very old” refers to adults aged ≥ 80 years (octogenarians and nonagenarians), whereas “older adults” refers to those aged ≥ 75 years when relevant. In this population, HF is frequently accompanied by multimorbidity, polypharmacy and geriatric syndromes, particularly frailty, which strongly influence prognosis, treatment tolerance and functional outcomes [1–4].

Frailty, defined as a state of reduced physiological reserve and increased vulnerability to stressors, is highly prevalent among older adults with HF and has been consistently associated with mortality, hospitalisation, impaired quality of life and treatment intolerance [1–4]. Beyond chronological age, frailty and multimorbidity shape the clinical trajectory of HF and challenge the direct application of guideline-based strategies derived largely from younger and more selected trial populations.

In this review, the term ‘heart failure’ is used with conceptual precision, distinguishing between symptoms/functional limitation, clinical congestion and left ventricular (LV) systolic dysfunction, as these may reflect distinct mechanisms in very old paced patients.

### Device-based therapies and CRT in very old adults

Device-based therapies, including permanent pacing and cardiac resynchronization therapy (CRT), are increasingly encountered in very old patients with HF. While CRT is an established treatment for selected patients with reduced ejection fraction and electrical dyssynchrony, its use in advanced age raises specific challenges. Older adults are underrepresented in pivotal CRT trials, often present with competing mortality risks, and may develop HF driven by device-related mechanisms such as right ventricular pacing–induced dyssynchrony rather than progressive cardiomyopathy alone [5–7].

### Limits of electrophysiology-driven decision-making in advanced age

In this context, CRT implantation or upgrade in very old adults cannot be approached solely through electrophysiological criteria. Decisions based exclusively on QRS duration, morphology or left ventricular ejection fraction may fail to capture key determinants of benefit and proportionality in advanced age.

Instead, CRT decision-making in very old patients requires an integrated cardiogeriatric perspective that considers physiological vulnerability, functional status, cognitive reserve, procedural burden and patient-centred goals. In advanced age, the expected benefit of CRT often relates more to symptom relief, functional recovery and quality of life than to survival prolongation.

### Rationale and scope

Despite the growing number of very old adults with HF and pacing systems, evidence guiding CRT use in this population remains limited. Existing evidence is largely derived from younger, selected trial populations and does not adequately capture the impact of frailty, functional reserve, cognitive vulnerability and competing risks that characterize advanced age. Moreover, HF in very old patients is frequently driven by potentially reversible device-related dyssynchrony, raising specific pathophysiological and decisional challenges not fully reflected in guideline-based algorithms.

This review aims to contextualise CRT decision-making in very old adults through a cardiogeriatric and physiology-based perspective, emphasizing proportionality of care and patient-centred outcomes. The objective is not to redefine guideline indications, but to support individualized clinical reasoning in advanced age and to highlight the relevance of geriatric dimensions in selecting patients most likely to derive meaningful benefit.

This narrative, concept-driven review is based on a targeted, non-systematic selection of peer-reviewed literature and clinical expertise, intended to contextualise CRT decision-making in very old adults rather than provide an exhaustive systematic synthesis. The literature informing this review was identified through targeted searches of PubMed and major cardiology journals between 2000 and 2025, using combinations of key terms including “cardiac resynchronization therapy,” “very old,” “octogenarians,” “frailty,” “pacing-induced cardiomyopathy,” and “conduction system pacing.” Selection focused on landmark randomised trials, large observational cohorts, contemporary guideline documents, and recent consensus statements relevant to advanced-age populations.

## Clinical illustration: pacemaker-induced heart failure in a very old patient

An 87-year-old patient with a history of permanent atrial fibrillation and high-grade atrioventricular conduction disease had previously undergone implantation of a single-chamber right ventricular pacemaker for symptomatic bradycardia. Over subsequent years, the patient developed progressive exertional dyspnoea, recurrent episodes of congestion and functional decline, leading to repeated hospitalisations for decompensated HF.

At reassessment, the patient had NYHA class III symptoms with recurrent congestion requiring three hospitalisations over the preceding 12 months. Device interrogation showed a right ventricular pacing burden of 96%, with a paced QRS duration of 178 ms. Echocardiography demonstrated LV systolic dysfunction (LVEF 32%, previously 58% three years earlier) with marked mechanical dyssynchrony, and secondary mitral regurgitation graded moderate-to-severe. Tricuspid regurgitation (TR) was systematically assessed and was moderate, predominantly functional in mechanism, without clear evidence of direct leaflet impingement by the pacing lead, acknowledging that lead-related TR may contribute to right-sided congestion in paced older adults.

Despite advanced chronological age and multiple comorbidities, the clinical presentation suggested a potentially modifiable pacing-related substrate (high right ventricular (RV) pacing burden with secondary LV dysfunction and dyssynchrony, while considering concomitant valve mechanisms), rather than irreversible myocardial disease. After multidisciplinary discussion incorporating cardiology and geriatric assessment—including evaluation of frailty, cognitive status, functional reserve and goals of care—a decision was made to pursue an upgrade to CRT.

Following CRT implantation, device checks confirmed a biventricular pacing percentage of 98% despite permanent atrial fibrillation. No atrioventricular nodal ablation was required in this pacing-dependent context. At 6-month follow-up, the paced QRS narrowed from 178 ms to 138 ms, NYHA functional class improved from III to II, and echocardiography showed partial recovery of LV systolic function (LVEF 32% to 40%) with reduced congestion and improved functional stability. This clinical course illustrates how, in selected very old patients, pacing-induced HF may represent a modifiable substrate and highlights the importance of integrating physiological mechanisms and geriatric dimensions when considering device-based therapies in advanced age.

## Pathophysiological rationale for CRT in older adults

### Right ventricular pacing–induced dyssynchrony and ventricular dysfunction

Chronic RV apical pacing induces an abnormal pattern of ventricular activation that mimics left bundle branch block, leading to electrical and mechanical dyssynchrony. This dyssynchrony alters myocardial strain distribution, promotes adverse left ventricular remodelling and impairs systolic efficiency, ultimately contributing to HF progression [8]. In older adults, who often exhibit reduced myocardial reserve and increased ventricular stiffness, the haemodynamic consequences of dyssynchrony may be particularly pronounced.

### Pacing-induced cardiomyopathy as a potentially reversible mechanism

Pacing-induced dyssynchrony refers to the electrical and mechanical asynchrony caused by chronic RV pacing. Pacing-induced cardiomyopathy (PICM) is a clinical entity in which this dyssynchrony is associated with the development of LV systolic dysfunction (i.e., a decline in LVEF), and should not be conflated with dyssynchrony in the absence of LV dysfunction. Pacing-induced cardiomyopathy is now a well-recognised entity, with observational studies reporting its occurrence in a substantial proportion of patients exposed to high RV pacing burdens [9]. The risk increases with cumulative pacing percentage and duration, and may manifest after years of apparently stable pacing. Importantly, this mechanism is potentially reversible, as correction of dyssynchrony through cardiac resynchronization therapy (CRT) can improve ventricular function and clinical status.

### Impact of dyssynchrony on secondary functional mitral regurgitation

Beyond global systolic impairment, RV pacing–induced dyssynchrony contributes to secondary functional MR by disrupting coordinated papillary muscle contraction and exacerbating annular dilatation. In older patients, who frequently present with pre-existing valvular degeneration or atrial functional MR, dyssynchrony may amplify regurgitant severity and precipitate decompensation. CRT has been shown to reduce functional MR through improved ventricular synchrony and reverse remodelling, thereby alleviating one of the key drivers of HF symptoms [10, 11].

### Device- and lead-related tricuspid regurgitation as a driver of congestion

In older paced patients, right-sided congestion may be driven or amplified by pacing- and lead-related TR. Trans-tricuspid leads can induce or worsen TR through leaflet impingement, tethering, or interference with coaptation, and pacing-related atrioventricular asynchrony may further exacerbate regurgitation. This mechanism is particularly relevant in advanced age, where atrial fibrillation and annular dilatation are common, and may explain congestion despite preserved LV systolic function. Therefore, systematic echocardiographic assessment of TR severity and mechanism after device implantation should be part of the cardiogeriatric evaluation, and TR should be considered alongside LV dyssynchrony when interpreting symptoms and selecting candidates for CRT upgrade or alternative pacing strategies.

Importantly, pacing-related dyssynchrony is most likely to result in clinically overt HF when it leads to or coexists with LV systolic dysfunction. In patients with preserved ejection fraction, dyssynchrony alone rarely explains decompensation; instead, severe haemodynamic substrates—particularly significant valvular disease such as TR—may represent the dominant driver of congestion. In this context, dyssynchrony should be interpreted as a potential contributor rather than the primary mechanism of HF in patients with preserved systolic function. Recent literature has also highlighted that HFpEF and HFmrEF presentations in acute HF frequently reflect alternative structural cardiac mechanisms rather than isolated diastolic dysfunction [12].

### Age-related myocardial vulnerability and limited compensatory reserve

Age-related myocardial changes—including increased fibrosis, impaired calcium handling and reduced β-adrenergic responsiveness—further limit the heart’s ability to compensate for dyssynchronous activation. Consequently, in very old adults, pacing-induced dyssynchrony may represent a disproportionately important and potentially modifiable contributor to HF, even when left ventricular ejection fraction is only moderately reduced. Alternative pacing strategies such as His bundle pacing have emerged as physiologically attractive options, but their feasibility and durability in very old, multimorbid patients remain variable, making CRT a relevant therapeutic consideration in selected cases [13].

Taken together, these mechanisms provide a strong physiological rationale for considering CRT upgrade in older adults with high RV pacing burden and HF symptoms, particularly when clinical deterioration is driven by potentially reversible dyssynchrony rather than advanced intrinsic myocardial disease.

### Alternative pacing strategies and their current limits in very old adults

Conduction system pacing strategies, including left bundle branch pacing, have emerged as physiologically attractive alternatives to biventricular pacing by preserving or restoring near-normal ventricular activation.

Recent evidence supports conduction system pacing (CSP) as a plausible alternative to conventional biventricular CRT in selected patients with CRT indications. Observational comparative data suggest that left bundle branch area pacing (LBBAP) can achieve effective electrical resynchronization with clinical outcomes that may be comparable to biventricular pacing in CRT candidates [14]. Small randomised studies have also reported non-inferiority of LBBAP/CSP compared with biventricular CRT with respect to echocardiographic and clinical response in systolic dysfunction and wide QRS [15, 16].

From a cardiogeriatric perspective, the potential relevance of CSP is that effective LV electrical resynchronization may be achieved using a single LBBAP lead, which in selected older patients could allow the use of a dual-chamber pacemaker instead of a conventional CRT system. This approach could reduce device hardware, potentially improve cost-effectiveness, and simplify procedures in frail patients; however, evidence in very old and frail populations remains limited, and careful individualized selection is required, particularly for upgrade procedures [17].

However, dedicated evidence in frail octogenarians (≥ 80 years) remains very limited, and most available data derive from younger or selected cohorts, which constrains geriatric generalizability. In this context, while conduction system pacing represents an important evolution in pacing physiology, its role at present in very old, multimorbid patients requires cautious evaluation within a cardiogeriatric framework.

## Evidence on cardiac resynchronization therapy in very old patients

### Evidence from randomised controlled trials: efficacy in selected populations

The efficacy of cardiac resynchronization therapy (CRT) has been firmly established in randomised controlled trials demonstrating reductions in mortality and HF hospitalisations, as well as improvements in symptoms and quality of life among selected patients with reduced ejection fraction and electrical dyssynchrony [18, 19]. However, these landmark trials enrolled predominantly younger and relatively robust populations, with limited inclusion of very old adults and minimal assessment of geriatric vulnerability.

Current guidelines recommend CRT in selected patients with HF, reduced LVEF and electrical dyssynchrony; however, these recommendations are largely derived from younger, non-frail populations. Notably, major international guidelines formulate CRT indications without specifying an upper age limit or age-based restriction; rather, they emphasize that candidacy should be individualized based on clinical context, comorbidity burden and expected benefit–risk balance.

### Underrepresentation of very old adults and limited geriatric assessment

In CARE-HF, participants had a mean age of 65 years (IQR 59–72), with 34% aged > 70 years, highlighting the limited representation of very old adults [18]. In COMPANION, age distributions similarly centered on the mid-to-late sixties (median 67 years [IQR 59–73] in an individual patient-data meta-analysis), and the proportion of patients aged ≥ 80 years is not clearly reported in these accessible trial summaries [19]. As a result, direct extrapolation of these outcomes to older adults (≥ 75 years), and especially to very old adults (≥ 80 years), remains uncertain, particularly in the presence of frailty or competing mortality risks. Notably, frailty, cognition, functional status, and geriatric outcomes were not systematically captured in pivotal CRT trials, limiting external validity in very old adults. Importantly, detailed age-stratified analyses for very old adults (≥ 80 years) are rarely reported in pivotal CRT trials, and available publications generally do not provide dedicated outcome data for this subgroup, further limiting extrapolation to advanced-age populations [18, 19].

### Observational and registry data in older populations

Observational studies and registry-based analyses provide complementary insights into CRT outcomes in older populations, but their interpretation is limited by heterogeneity in baseline risk, follow-up duration, and the potential for referral and survivor selection. To facilitate applicability appraisal, we highlight key cohort characteristics (sample size, age distribution, proportion ≥ 80 years when available, follow-up and main endpoints). In a retrospective single-centre cohort of 728 consecutive CRT recipients implanted between 2002 and 2008, 90 patients (12.4%) were aged > 80 years; octogenarians and younger patients showed similar post-CRT improvements in NYHA class, LVEF, and mitral regurgitation severity. The overall 30-day complication rate was 12.2% with no significant difference by age group, while crude survival was worse in octogenarians but not significantly different after multivariable adjustment (HR 1.23, 95% CI 0.83–1.84; *p* = 0.31) [20]. Overall, available cohorts suggest that selected older adults—including octogenarians—can derive meaningful symptomatic and functional benefit after CRT, whereas survival gains may be attenuated by competing risks; procedural success is generally high, but older patients may experience more non-cardiac complications and longer recovery.

### CRT response in pacing-induced cardiomyopathy and device-related dyssynchrony

Notably, pacing-induced cardiomyopathy (with LVEF decline) and pacing-related dyssynchrony in patients with high RV pacing burden have been associated with a favourable response to CRT across age groups, including in elderly patients [21].

Although randomised data specifically in very old adults are scarce, controlled evidence supports CRT (or BiV pacing) as a strategy to mitigate RV pacing–related deterioration in populations requiring substantial ventricular pacing. In BLOCK-HF, biventricular pacing was superior to conventional RV pacing in patients with atrioventricular block and LV dysfunction (LVEF ≤ 50%), with fewer composite events (death, urgent HF care, or adverse LV remodelling) and a reported LV lead–related complication rate of 6.4% [22]. More recently, randomised upgrade trial designs have specifically targeted patients with pre-existing devices, wide paced QRS, reduced LVEF, and substantial RV pacing burden, providing a directly relevant evidence framework for upgrade decisions—even if generalizability to frail octogenarians remains uncertain [23].

In such contexts, CRT addresses a clearly identifiable and potentially reversible mechanism of HF, which may partly explain the preserved clinical response observed in advanced age.

### Functional and quality-of-life outcomes as primary endpoints in advanced age

Quality-of-life outcomes represent a particularly relevant endpoint in very old adults. When reported, they are assessed using validated patient-reported outcome measures such as the Minnesota Living with Heart Failure Questionnaire (MLHFQ) and, less consistently, generic health-status instruments (e.g., EQ-5D) (24,25). Symptom burden and functional status are also frequently captured through NYHA functional class and related clinical endpoints, but these should be interpreted alongside patient-reported measures when the primary objective is meaningful functional benefit rather than survival extension. Overall, available data indicate that CRT can improve symptoms, functional class, and perceived well-being even when mortality effects are modest or difficult to demonstrate in advanced age [24, 25]. These findings underscore the importance of prioritising functional and patient-centred outcomes when assessing CRT value in geriatric populations, where multimorbidity and competing risks may attenuate survival gains.

### Limits of available evidence and implications for individualized interpretation

Overall, the available evidence suggests that while very old patients are underrepresented in randomised trials, selected individuals may derive meaningful symptomatic and functional benefit from CRT. However, the heterogeneity of ageing trajectories and the influence of frailty, multimorbidity and competing risks necessitate individualized interpretation of trial data rather than uniform application of guideline criteria.

However, these observational data are inherently subject to selection bias, as very frail patients and those with limited life expectancy are less likely to be referred for CRT.

## Geriatric dimensions influencing CRT decision-making

### Frailty as a central determinant of benefit and tolerance

In very old adults, decision-making regarding CRTextends beyond conventional electrophysiological criteria and requires careful consideration of geriatric domains that strongly influence outcomes and proportionality of care. Frailty, functional status, cognitive impairment and multimorbidity are particularly relevant, as they modulate both the potential benefit and the risks associated with device-based interventions.

Frailty has emerged as a central determinant of prognosis and treatment tolerance in cardiovascular disease. It reflects reduced physiological reserve and increased vulnerability to stressors, including invasive procedures and peri-procedural complications. In older patients with HF, frailty has been consistently associated with higher mortality, increased hospitalisation rates and poorer functional recovery, independent of chronological age [26]. Importantly, frailty is not systematically assessed in CRT trials, limiting the ability to stratify benefit in very old populations.

### Procedural burden and peri-procedural vulnerability in advanced age

Beyond physiological eligibility, the procedural burden of CRT deserves particular consideration in very old adults. CRT implantation or upgrade is a longer and more complex intervention than conventional pacemaker implantation, often involving venous access challenges, coronary sinus cannulation and prolonged fluoroscopy time. Although most procedures can be performed under local anaesthesia with conscious sedation, very old patients may be more vulnerable to peri-procedural stress, delirium, haemodynamic instability and post-procedural functional decline. These factors highlight the importance of assessing procedural tolerance, anaesthetic risk and anticipated recovery when considering CRT in advanced age, particularly in frail patients. These considerations should be incorporated upstream into the decision-making process, rather than being viewed solely as peri-procedural risks.

In addition, CRT upgrade procedures carry specific and clinically meaningful risks that are particularly relevant in very old, multimorbid patients. The risk of cardiac implantable electronic device (CIED) infection is higher than in de novo implants and may be especially consequential in frail individuals, as device-related infection is associated with substantial morbidity and mortality, frequently requires prolonged antibiotic therapy and complex reinterventions (including system extraction in many cases), and may lead to major functional decline. Beyond infection, upgrade procedures entail additional practical vulnerabilities: pocket haematoma—particularly in the context of anticoagulation for atrial fibrillation—may increase short-term morbidity and downstream infection risk; venous stenosis or occlusion can limit vascular access for additional leads; and lead management (abandoned leads versus extraction) may need to be considered, with extraction carrying higher procedural risk and generally being avoided unless clinically necessary. Coronary sinus cannulation may fail or require contrast exposure, which can be problematic in patients with renal vulnerability. Furthermore, CRT is associated with a non-response rate of approximately 20–30% in selected populations, meaning that even technically successful implantation may not translate into meaningful clinical improvement in patients with limited physiological reserve. These upgrade-specific risks should therefore be explicitly discussed during shared decision-making and incorporated upstream into proportionality-based cardiogeriatric selection [27].

### Functional status and patient-centred goals of care

Functional status and autonomy are equally critical considerations. Preservation of mobility, independence in activities of daily living and symptom control often represent primary goals of care for older adults, sometimes outweighing survival extension. CRT may offer meaningful improvements in functional capacity and quality of life in selected patients, but these benefits may be attenuated or absent in individuals with advanced functional dependence or limited baseline reserve [28].

### Cognitive vulnerability and shared decision-making

Cognitive impairment further complicates CRT decision-making by affecting adherence to follow-up, understanding of device-related care and the capacity to engage in shared decision-making. Although rarely captured in cardiology trials, cognitive vulnerability is common in very old patients with HF and must be incorporated into a comprehensive assessment of anticipated benefit and burden [29].

### Multimorbidity, proportionality and individualized selection

Finally, multimorbidity and polypharmacy influence both procedural risk and long-term management. Competing non-cardiac conditions may limit life expectancy or functional recovery, thereby reducing the relative value of CRT in some patients. In this context, proportionality of care and alignment with patient priorities become central principles. Rather than applying uniform eligibility criteria, CRT decisions in very old adults should be individualized, integrating geriatric assessment to identify patients in whom correction of dyssynchrony is likely to translate into meaningful functional gain [30].

## A cardiogeriatric decision-making framework for CRT

### Limits of guideline-based decision-making in advanced age

Given the limited representation of very old adults in randomised trials and the absence of systematic geriatric assessment in CRT studies, a cardiogeriatric decision-making framework is required to translate trial-derived evidence into advanced-age clinical practice. Such a framework does not replace guideline indications but complements them by incorporating physiological vulnerability, functional reserve and patient-centred goals.

International HF and pacing guidelines acknowledge the importance of individualized decision-making in older adults, particularly in the presence of multimorbidity and frailty [31, 32]. Importantly, these recommendations are not framed with an age cut-off, reinforcing the need for individualized decision-making in advanced age. However, these documents provide limited operational guidance on how geriatric dimensions should be integrated into CRT selection. As a result, CRT implantation in very old patients is often guided by electrophysiological criteria alone, despite substantial heterogeneity in ageing trajectories.

### Identification of a potentially reversible physiological substrate

A cardiogeriatric framework for CRT begins with identification of a potentially reversible mechanism of HF, such as pacing-induced dyssynchrony with secondary LV systolic dysfunction (pacing-induced cardiomyopathy), secondary functional MR, or pacing/lead-related TR contributing to right-sided congestion. In this context, CRT targets a clearly defined physiological substrate rather than progressive, diffuse myocardial disease. This distinction is particularly relevant in advanced age, where the likelihood of meaningful functional improvement depends on reversibility rather than disease severity alone. In selected patients, conduction system pacing may also represent an alternative strategy to achieve resynchronization, particularly when minimizing device complexity and procedural burden is a priority in frail older adults.

### Integration of geriatric assessment and proportionality of care

The second step involves structured geriatric assessment, including evaluation of frailty, functional status, cognition, comorbidity burden and life expectancy. In practice, this can rely on pragmatic tools such as the Clinical Frailty Scale, gait speed or chair-rise performance, ADL/IADL dependency, and brief cognitive screening (e.g., Mini-Cog or MoCA), which directly inform procedural proportionality (tolerance to intervention, recovery capacity, follow-up feasibility) and help structure the device conversation (symptom relief versus survival goals). Patients who are non-frail or mildly frail, retain functional reserve and express goals aligned with symptom relief and functional improvement may reasonably be considered for CRT, even at advanced chronological age. Conversely, in patients with advanced frailty, severe cognitive impairment, or HF driven by diffuse myocardial disease rather than dyssynchrony, CRT is unlikely to provide meaningful benefit despite formal guideline eligibility.

### Shared decision-making and patient-centred goals

Finally, shared decision-making is central to this framework. Discussions should explicitly address expected benefits—primarily symptomatic and functional rather than survival-related—as well as procedural risks (including upgrade-specific device infection risk), follow-up burden and device-related implications. Incorporating patient values and preferences is essential to ensure that CRT aligns with individual goals of care, particularly in very old adults [33].

### Conceptual synthesis

This cardiogeriatric framework emphasizes that CRT in advanced age is not a binary decision based solely on guideline thresholds, but a nuanced, individualized process that balances physiological reversibility, geriatric vulnerability and patient-centred priorities.

**Fig. 1:**
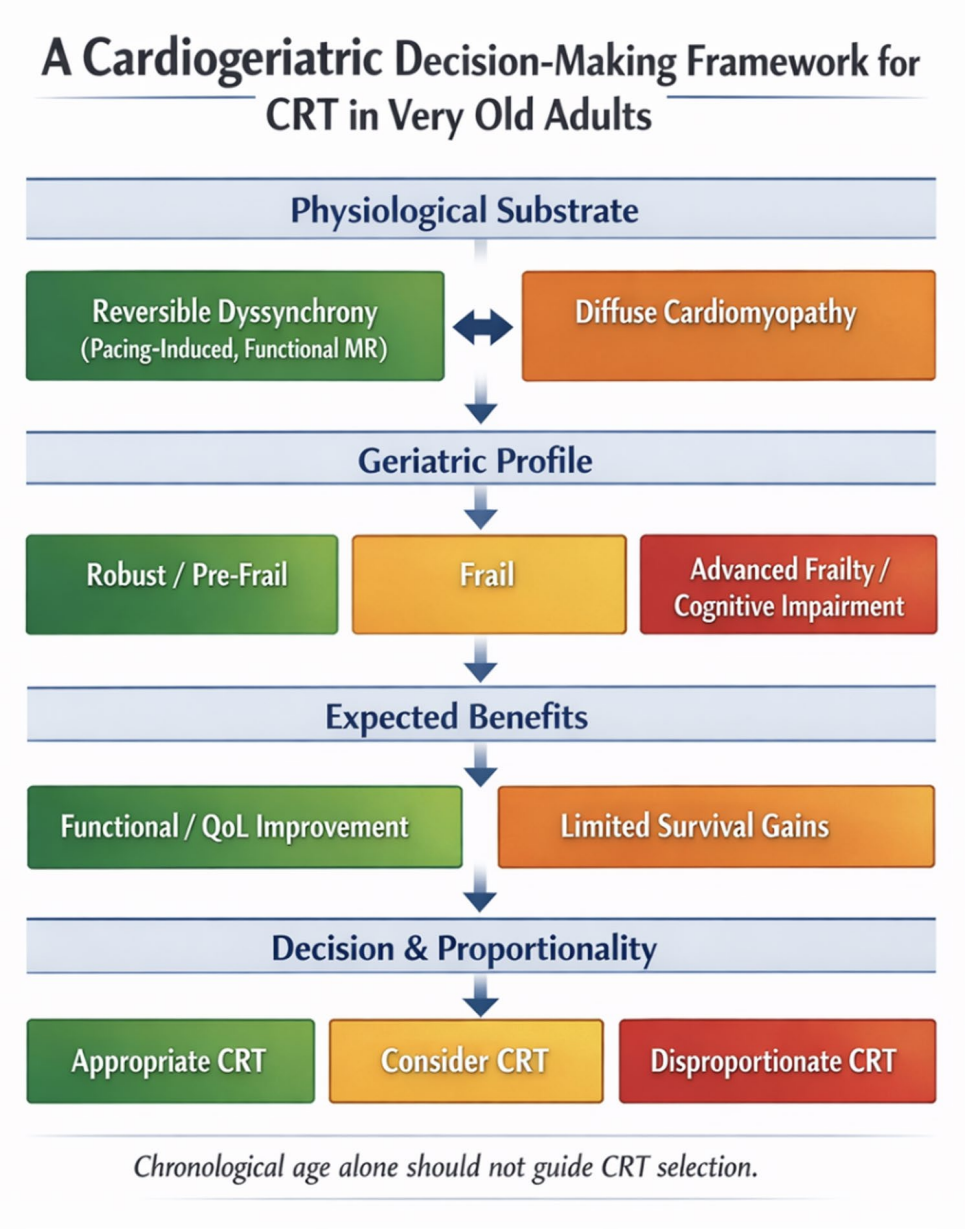
A cardiogeriatric decision-making framework for cardiac resynchronization therapy in very old adults. Legend: This figure presents a cardiogeriatric framework for cardiac resynchronization therapy (CRT) in very old adults with heart failure (HF), integrating the underlying pathophysiological substrate, geriatric vulnerability (e.g., Clinical Frailty Scale, ADL/IADL assessment, Mini-Cog or MoCA), and patient-centred goals of care. By aligning expected benefit with physiological reversibility and proportionality of care, the framework supports individualized CRT decision-making beyond chronological age or electrophysiological criteria alone

A conceptual overview of this cardiogeriatric decision-making framework is presented in Fig. 1.

## Discussion

### Beyond trial-derived criteria: the need for individualized reasoning in advanced age

This narrative review highlights the complexity of CRT decision-making in very old adults with HF and underscores the limitations of applying trial-derived criteria to heterogeneous geriatric populations. This framework is not intended to redefine indications for CRT, but to support individualized clinical reasoning in the absence of geriatric-representative trial data. While CRT is supported by robust evidence in selected patients, the clinical scenarios encountered in advanced age—characterized by multimorbidity, frailty and competing risks—require a broader interpretative framework that integrates physiological mechanisms and geriatric principles.

### Physiological reversibility as a key determinant of benefit

The illustrative case presented here exemplifies a situation in which HF progression was largely driven by pacing-related mechanisms (including a high RV pacing burden with secondary LV dysfunction and dyssynchrony, and the potential contribution of pacing-related valve dysfunction), a potentially reversible mechanism. In such contexts, CRT targets a clearly identifiable pathophysiological substrate rather than diffuse myocardial disease. This distinction is crucial in very old patients, as the likelihood of meaningful benefit depends less on chronological age than on reversibility, functional reserve and proportionality of care. When dyssynchrony appears to be a major contributing mechanism, CRT may yield clinically relevant improvements even in advanced age.

### Competing risks and outcome prioritisation in very old adults

However, the potential benefits of CRT in older adults must be weighed against competing outcomes. In very old populations, non-cardiovascular mortality and functional decline often compete with traditional HF endpoints. As a result, survival gains may be modest or absent, whereas improvements in symptoms, functional capacity and quality of life become the primary outcomes of interest. This shift in priorities aligns with contemporary frameworks for decision-making in advanced HF, which emphasize proportionality, shared decision-making and alignment with patient goals rather than uniform escalation of device-based therapies [34]. In this context, the absence of survival benefit should not be interpreted as therapeutic failure, but rather as a reflection of outcome prioritisation in advanced age.

### The unmet need for geriatric assessment in CRT decision-making

The integration of geriatric assessment into CRT decision-making remains a major unmet need. Frailty, cognitive impairment and functional dependence—rarely assessed in CRT trials—are strong predictors of adverse outcomes and treatment intolerance. Their absence from pivotal studies limits external validity and contributes to uncertainty when extrapolating evidence to very old patients. Incorporating geriatric dimensions into clinical reasoning does not imply withholding effective therapies, but rather refining patient selection to maximize benefit and avoid disproportionate interventions [35].

### Ethical considerations and shared decision-making

From an ethical perspective, CRT in advanced age should be framed within a model of individualized, patient-centred care. Shared decision-making is particularly important, as device implantation carries long-term implications for follow-up, procedural burden and end-of-life care. Early integration of palliative principles and explicit discussion of goals of care may help ensure that CRT aligns with patient values, especially when life expectancy is limited or functional decline predominates [36].

### Implications for research and future directions

Finally, this review reinforces the broader issue of underrepresentation of very old adults in cardiovascular trials. Despite being the population most affected by HF, octogenarians and frail individuals remain systematically excluded from randomised studies, perpetuating a gap between evidence generation and real-world practice. Addressing this gap will require not only inclusive trial designs but also greater incorporation of geriatric outcomes and functional endpoints that reflect what matters most to older patients [37].

Until such data become available, cardiogeriatric reasoning remains essential to ensure proportional and patient-centred use of CRT in advanced age. Future studies specifically designed to include very old adults and to integrate frailty, functional status and patient-centred outcomes into device trials are needed to better inform CRT decision-making in ageing populations.

## Conclusion

In very old adults with HF, CRT should be understood primarily as a cardiogeriatric decision rather than the mechanical application of electrophysiological guideline criteria.

As illustrated by pacing-induced HF, CRT may provide meaningful symptomatic and functional benefit in selected older patients when HF is driven by a potentially reversible mechanism, even in advanced age. In this setting, the relevance of CRT depends less on traditional electrical thresholds than on physiological reversibility, preserved functional reserve and alignment with patient priorities. Conversely, in the presence of advanced frailty or limited life expectancy, CRT may represent a disproportionate intervention despite formal eligibility.

A cardiogeriatric, physiology-based approach enables clinicians to balance expected benefit against vulnerability and to prioritize outcomes that matter most to older adults, such as symptom relief, maintenance of autonomy and quality of life. Ultimately, CRT in very old patients should be considered not as a binary, guideline-driven indication, but as an individualized therapeutic choice informed by geriatric assessment and shared decision-making.

## Data Availability

No datasets were generated or analysed during the current study.
